# Identification of founder and novel mutations that cause congenital insensitivity to pain (CIP) in palestinian patients

**DOI:** 10.1186/s12920-023-01544-5

**Published:** 2023-05-30

**Authors:** Boushra Khaled, Mahmoud Alzahayqa, Ahmad Jaffal, Husam Sallam, Rua’a Thawabta, Mamoun Mansour, Akram Alian, Zaidoun Salah

**Affiliations:** 1 Molecular Genetics and Genetic Toxicology Program, Arab American University, Ramallah, Palestine; 2 Molecular Genetics Lab, Medicare Laboratories, Ramallah, Palestine; 3 Sana Medical Center, Jerusalem, Palestine; 4 Al-makassed Islamic Charitable Hospital, Jerusalem, Palestine; 5 Department of Biochemistry, University of Utah School of Medicine, Salt Lake City, UT USA

**Keywords:** Pain, CIP, CIPA, NTRK1and SCN9A

## Abstract

**Background:**

Congenital insensitivity to pain (CIP) is a rare autosomal recessive disorder characterized primarily by an inability to perceive physical pain from birth, resulting in the accumulation of bruising, inflammation, and fractures that affect patient’s life expectancy. CIP has different forms including CIP and CIPA. CIP with Anhidrosis (CIPA) is the most common type of CIP, which is caused mainly by mutations in *NTRK1* and *NGF* genes, and is characterized by mental retardation and the inability to sweat (Anhidrosis). Because of high consanguinity rates in Palestine, this rare disease appears to have a higher frequency than in other communities. However, there were no systematic studies to address the genetic factors that cause CIP in the Palestinian community.

**Methods:**

In our study, we used Sanger and Whole exome sequencing to genotype members of five CIP-affected Palestinian families.

**Results:**

Our results confirm the presence of the founder c.1860-1861insT mutation in the *NTRK1* gene of Palestinian Bedouin CIPA patients. Furthermore, one CIPA family carried a missense c.2170 G > A (G724 S) mutation in exon 16 of the *NTRK1* gene. Finally, a novel nonsense c.901 A > T mutation (K301*) was detected in exon 7 of the *SCN9A* gene in CIP without anhidrosis family.

**Conclusions:**

Our study revealed three mutations that cause CIP and CIPA in the Palestinian community, which can help in improving the process of diagnosis and genetic counseling and establishing protocols for the diagnosis and follow-up for the affected individuals. This is especially important given that early diagnosis and medical care interference can prevent unpleasant CIP and CIPA complications.

**Supplementary Information:**

The online version contains supplementary material available at 10.1186/s12920-023-01544-5.

## Introduction

Pain is an unpleasant physical and emotional drain with great survival value. Specialized neurons of pain, called nociceptors, can early alarm the organism of potentially dangerous contacts and thus help in preventing tissue damage [1]. Different conditions can lead to pain loss, including diabetes, stroke, tumors, infections, as well as genetic factors. Congenital Insensitivity to Pain (CIP) is a very rare autosomal recessive genetic disorder that leads to insensitivity to pain. CIP is characterized by losing the ability to perceive physical pain from birth [2], which leads to deaths due to the inability to detect injuries and illness [3]. CIP is of two types: The first is Hereditary Sensory and Autonomic Neuropathy (HSAN), which is characterized by the inability of nociceptors to develop, or even their death, due to the absence of trophic signals. HSAN4 is the most common type of HSAN that is caused by mutations in Nerve Tropomyosin Receptor Kinase A (*NTRK1*) gene [4]. The second type of CIP is characterized by the inability to activate tissue damage signals and the inability to smell (anosmia). This type is mostly related to mutations in Sodium voltage-gated Channel Alpha subunit 9 (*SCN9A*) gene, which encodes for the formation of the alpha subunit of Nav1.7 Sodium channels found in nociceptors and olfactory sensory neurons [1, 5].

Due to the inability of perceiving physical pain, CIP patients suffer from the accumulation of infections, fractures, bruises and wounds that affect their life expectancy, making this disorder of an important concern to physicians around the world. In addition to the common features of CIP, which include the inability to perceive pain, self-mutilating behaviors, and musculoskeletal manifestation, other special characteristics, such as unexplained fever, reduced sweating and mental retardation, are associate with a condition called CIP with Anhidrosis (CIPA) [4]. CIPA is correlated with almost 100 mutations in the *NTRK1* gene [6], which is essential for the survival of neurons in the brain during embryonic development. Moreover, NTRK1 has a major role in regulating the level of peptide neurotransmitters/neuromodulators in the mature sympathetic and sensory neurons [6], which is important for the development and proper function of these cells. NTRK1 is also involved in immune, inflammatory and stress responses following tissue damages.

CIP has variable prevalence and different genetic basis in different ethnic groups (reviewed in [7]). Various worldwide studies have shown that the majority of CIP cases are caused by different mutations in the *SCN9A* gene [8]. Interestingly, studies in Palestine nearby countries showed that CIPA patients mainly carry mutations in the *NTRK1* gene, indicating founder effects [9–12]. Because CIP and HSAN are autosomal-recessive disorders, they are more prevalent in areas with high consanguinity [7]. Thus, because of the high consanguinity rates, it is plausible to see higher frequency for CIP in Palestine than in other nearby countries. Moreover, the diagnosis of most CIP cases in Palestine depend on the clinical picture instead of relying on genetic testing [6]. Therefore, we conducted this study, the first of its kind in the Palestinian community, to investigate five CIP families with several affected individuals. We aimed to identify CIP causing mutations in an effort to aid the development of testing protocols and to provide better genetic counselling for the affected families in Palestine. While our study confirms the presence of previously identified mutations in *NTRK1* gene, we reveal a novel mutation in the *SCN9A* gene.

## Materials and methods

All methods were performed in accordance with the relevant guidelines and regulations of AAUP ethical committee.

### Study subjects and medical history

The study involved five unrelated Palestinian families. The eight affected family members were examined by orthopedic doctor and were diagnosed with CIP. Six of the patients were diagnosed as CIPA patients and two of them as CIP without anhidrosis. Members of AP01, AP02, AP03 and AP04 families suffered from lack of pain sensation, increased body temperature, self-mutilation like finger amputation, mental retardation, recurrent infections, avascular necrosis and anhidrosis. Thus, affected members of these families were diagnosed as CIPA cases. For AP05 family, the affected members were diagnosed as CIP patients (without anhidrosis) because they suffered from insensitivity to pain, infections and Anosmia. The study was approved by AAUP ethical committee and all patients and their families signed a consent form to approve their willingness to participate in the study.

### Patient genotyping

For genotyping and variant segregation, primers were designed to amplify the 17 exons of *NTRK1* and exon 7 of *SCN9A* genes including intronic regions flanking the different exons (Supplementary Table 1). The primers were designed by using primer3 software http://primer3.ut.ee/. The reference sequence used for variant annotation was *NTRK1* mRNA sequence with accession number NM_002529.4.

### DNA extraction and PCR amplification

DNA was extracted from 300ul of peripheral blood by following Wizard***®*** genomic DNA purification kit (Promega, USA) protocol. The DNA concentration and purity were measured by using NanoDrop 2000 (Thermo scientific). Amplification of DNA fragments was carried out in 20 µL reaction mix that contained 10 µL PCR Master mix (Go Tag®, Promega, USA), 3 µL of DNA (~ 100 ng), 0.5 µM of each forward and reverse primers (Supplementary Table 1), and 5 µL nuclease free water. The PCR reaction was then run on Thermal cycler (Flex Cycler, analytikjena) using the following program; Initial denaturation at 95 °C for 5 min followed by 34 cycles of denaturation at 95 °C for 20 s, annealing at 57.6 °C (for NTRK1 fragments) and at 56 °C (for SCN9A fragment) for 30 s, and extension step at 72 °C for 1 min followed by a final extension step for 10 min at 72 °C. PCR products were visualize by using UV transilluminator (Chemi Doc, BIO RAD).

### Sanger sequencing

PCR products were first cleaned using EPPIC Fast cleaning kit (A&A Biotechnology) according to the manufacturer’s instructions. In Brief, 1 µL of EEPIC Fast enzymatic solution was added to 5 µL of PCR product and placed in thermal cycler at 37 °C for 10 min, followed by 1 min at 80 °C. Sanger sequencing was done by using BigDye Terminator v3 kit (Applied biosystem) according to manufacturer’s instructions. Sequencing was done using Applied Biosystems Genetic analyzer 3500 (Applied Biosystems).

### Whole exome sequencing

DNA library was first prepared from 500ng of genomic DNA by using Illumina® DNA Prep with Enrichment kit (Illumina, Inc) following the manufacturer’s instructions. DNA libraries were sequenced using NextSeq 5500 (Illumina, Inc). The collected data was then aligned to the reference human genome (hg19) using BWA aligner. Mapped reads (BAM format) were then processed and PCR duplicates were removed, base quality was recalibrated and areas around indels were realigned. Then, variant annotation was carried out using ANNOVAR (http://annovar.openbioinformatics.org/). Possible pathogenic variants were then filtered according to their frequency by using different databases including gnomAD, and PopFreqMax, and according to their predicted pathogenicity by using SIFT, PolyPhen2, REVEL, Mutation taster, Proven and FATHMM software. Finally, we selected the variants that correlates with the clinical findings of the patient and segregated in the family.

### Structure modeling and analysis

PyMOL, Molecular Graphics System, Schrödinger, LLC, was used to fetch NTRK1 reported structures, superimpose the various structures, measure distances, perform local amino acid substitutions, and prepare the images. Alphafold2 structure prediction was performed using the ColabFold platform [13] using 498–796 fragment of NTRK1 with Ser substituting Gly724 in the sequence. Structures preserving the NTRK1 fold but containing natural substitutions at position 724 were identified by searching the NTRK1 sequence against structure databank using the XtalPred server [14].

## Results

### Family description and clinical features

**Fig. 1:**
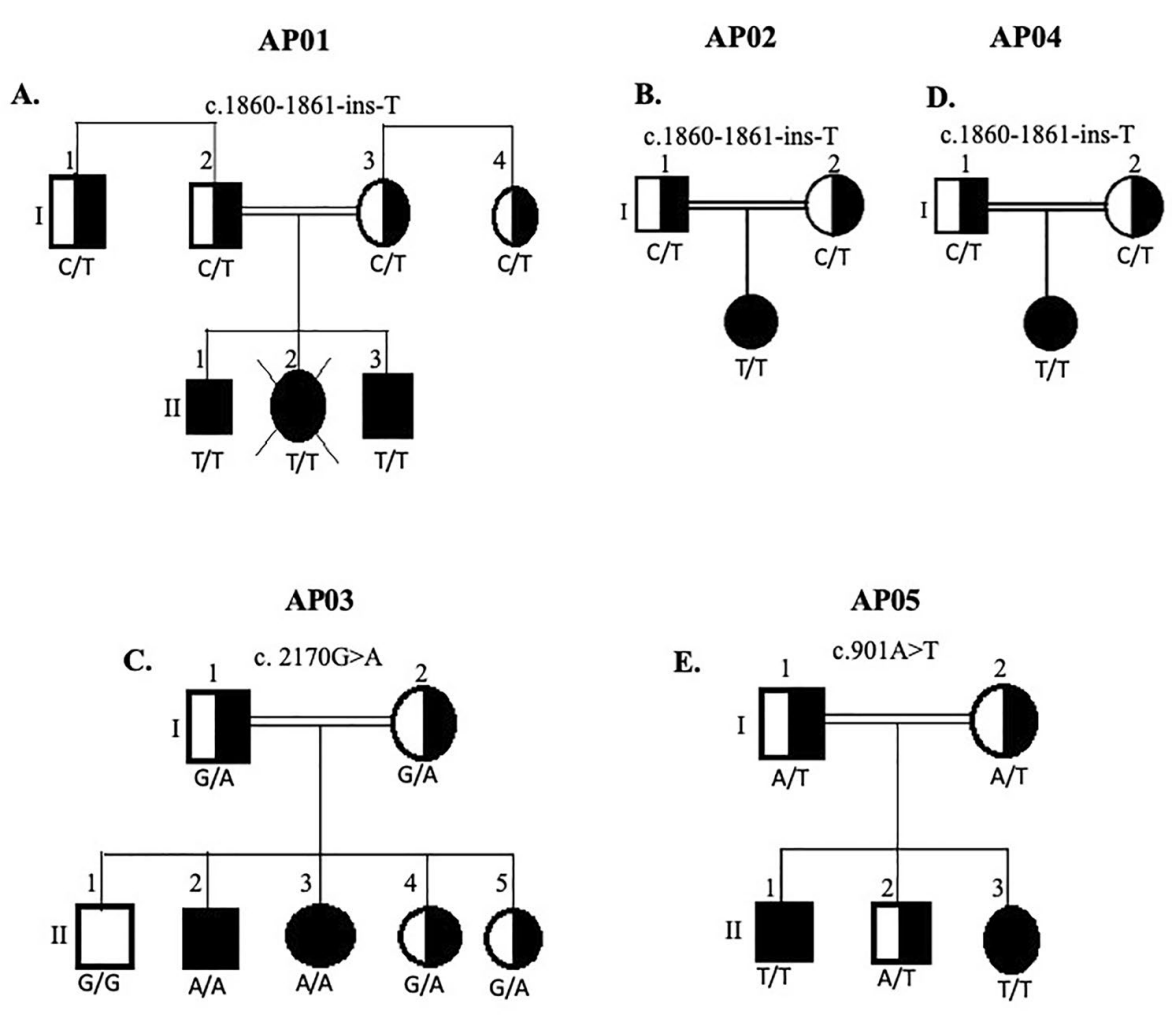
**Family pedigrees and genotypes. (A)** family AP01 **(B)** Family AP02 **(C)** Family AP03 **(D)** Family AP04 **(E)** Family AP05. Parallel lines indicate consanguinity. Black square means Homozygous affected male. Black circle means Homozygous affected female. Half white half black squares and circles represents Heterozygous males and females respectively

This research involved five unrelated Palestinian families, all of which were products of consanguineous marriages (Fig. 1). First, we performed clinical examination for the eight patients and their family members who participated in this study. The findings of the clinical examination of all participants are summarized in Table 1. Six of the patients suffered from inability to perceive physical pain, unexplained fever, mild mental retardation, and inability to sweat, therefore, they were diagnosed as CIPA patients. These CIPA affected family members also suffered from self-mutilation and infections, and some of them suffered from avascular necrosis (Table 1). The last two patients from AP05 family had mixed features where they were unable to perceive physical pain albeit they were mentally normal and able to sweat. One of these two patients was also unable to smell. The affected members of this family were, therefore, diagnosed with CIP without anhidrosis.

### Two mutations identified in CIPA patients

**Fig. 2:**
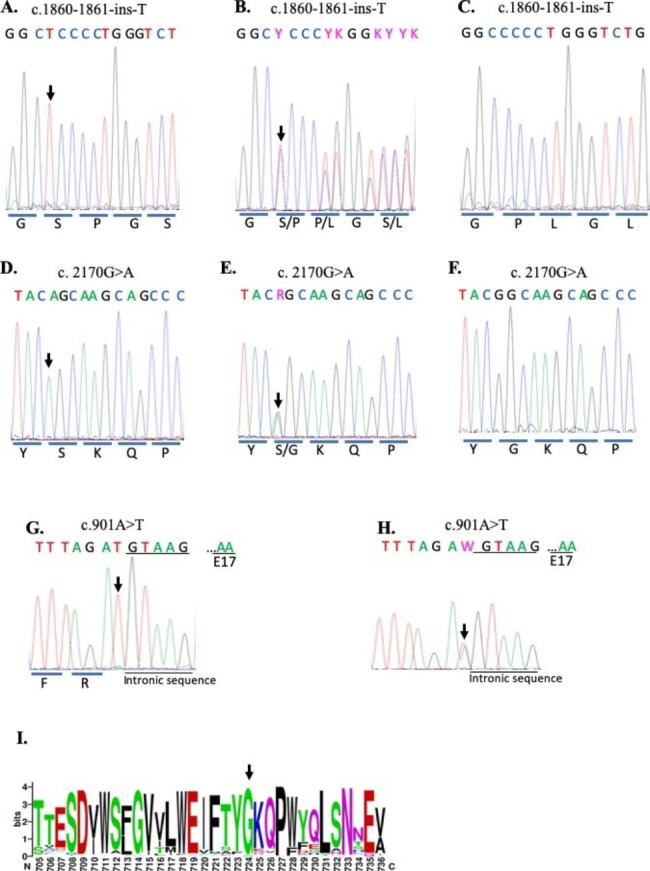
**Sanger sequence analysis of affected gene sequences.** Representative electrophergram results of partial sequence of exons 15 and 16 of *NTRK1* and exon 7 of SCN9A. **A.** Homozygous c.1860-1861insT mutation in *NTRK1* in the affected proband **B.** Heterozygous c.1860-1861insT mutation in the family members. **C.** Wild type normal control for c.1860-1861insT mutation. **D**. Homozygous c.2170 G > A mutation in *NTRK1* gene in the affected proband **E.** Heterozygous c.2170 G > A mutation in the family members. **F.** Wild type normal control for c.2170 G > A mutation. **G**. Homozygous c.901 A > T mutation in the *SCN9A* gene in affected proband **(H)** Heterozygous c.901 A > T mutation in the family members. Letters under the electropherograms represent the amino acids encoded by the sequenced area of the genes. Arrows indicate the mutated nucleotide. **(I)** Amino acid conservation of NTRK1 protein segment surrounding G724. Conservation predicted using Gremlin webserver [25] and figure was generated using WebLogo [26]

Six of the studied patients suffered from CIPA (Table 1), which is usually related to mutations in *NTRK1* gene. Hence, to identify the pathogenic variants in these patients, we initially pooled the DNA samples of all CIPA patients and Sanger sequenced all *NTRK1* gene exons. This primary analysis revealed the presence of two mutations in the *NTRK1* gene: a frameshift mutation in exon 15 (c.1860-1861insT) (Pro621Ser fsTer12) and a missense mutation in exon16 (c.2170 G > A) (p. Gly724 Ser). Next, the DNA of each patient, individually, was tested for these two mutations. The analysis revealed that the affected subjects from AP01, AP02 and AP04 families are homozygous (T/T) for the (c.1860-1861insT) mutation (Figs. 1A, B, and D and 2A). The insertion of the T base pair at this position leads to the formation of a pre-mature termination codon resulting in a truncated NTRK1 variant lacking a functional kinase domain. CIPA patients from AP03 family were homozygous (A/A) for (c.2170 G > A) mutation (Figs. 1C and 2D), which substitutes the original glycine residue 724 with serine (p. Gly724 Ser).

To further demonstrate that the identified mutations are the ones responsible for CIPA in the studied families, we performed segregation analysis for these mutations. We genotyped all participating family members for the identified mutations by Sanger sequencing. As shown by a representative electropherogram in Fig. 2B, all parents in AP01, AP02 and AP04 families were heterozygous (C/T) for the c.1860-1861insT mutation. For AP01 family, one female sibling died because of the disease complications. Moreover, the family members I1 and I4 of AP01 family were referred to us for testing because they wanted to marry each other. As indicated in Fig. 1A, they are heterozygous for c.1860-1861insT mutation and thus they were further referred for medical consultation before they marry.

**Table 1:** Characteristics and medical history of CIP families

Family code	Patient code	Age year	Sex	CIP	CIPA	Phenotypic features	Mental Retardation	Self mutilation	infections	Avascular Necrosis
AP01	AP01-01	42	M	-	-	-	-	-	-	-
	AP01-02	38	F	-	-	-	-	-	-	-
	AP01-03	18	M	-	+	Anhidrosis	+	+	+	+
	AP01-04	17	M	-	+	Anhidrosis	+	+	+	+
	AP01-05	23	F	-	-	-	-	-	-	-
	AP01-06	24	M	-	-	-	-	-	-	-
AP02	AP02-01	-	M	-	-	-	-	-	-	-
	AP02-02	-	F	-	+	Anhidrosis	+	+	+	+
AP03	AP03-01	47	M	-	-	-	-	-	-	-
	AP03-02	46	F	-	-	-	-	-	-	-
	AP03-03	23	M	-	-	-	-	-	-	-
	AP03-04	20	F	-	-	-	-	-	-	-
	AP03-05	18	F	-	+	Anhidrosis	+	+	+	-
	AP03-06	16	M	-	+	Anhidrosis	+	+	-	-
	AP03-07	11	F	-	-	-	-	-	-	-
AP04	AP04-01	-	M	-	-	-	-	-	-	-
	AP04-02	12	F	-	+	Anhidrosis	+	+	+	+
AP05	AP05-01	34	M	-	-	-	-	-	-	-
	AP05-02	28	F	-	-	-	-	-	-	-
	AP05-03	8	F	+	-	Anosmia	-	-	+	-
	AP05-04	7	M	+	-	Anosmia	-	-	+	-
	AP05-05	4	M	-	-	-	-	-	-	-

In AP03 family, mutation segregation analysis showed that the parents and unaffected siblings are heterozygous for c. 2170G > A mutation (Fig. 2E), confirming that this is the CIPA causative mutation in this family. A sample from normal unaffected subject was used to confirm the absence of c.1860-1861insT and c. 2170G > A mutations (Fig. 2C F respectively). Altogether, these results demonstrate that c.1860-1861insT is the CIPA causative mutation in AP01, AP02 and AP04 families, whereas c. 2170G > A is the causative mutation in AP03 families.

### *In silico* analysis and ACMG criteria of c. 2170G > A mutation

Whereas the c. 2170G > A mutation has previously been identified in nearby communities [9, 12], its identification in the Palestinian community is unique to our study. Still, the pathogenicity and underlying molecular basis of this variant are yet to be explored. Therefore, we employed various *in silico* tools to analyze this mutation. We first assessed the conservation level of the amino acid glycine at this position and found it highly conserved (Fig. 2-I & Suppl. Figure 1). Next, we analyzed the pathogenicity of this mutation using various tools including Polyphen, SIFT, PhD-SNP, mutation taster, Clinvar, and Mutpred. In addition, we addressed the variant pathogenicity according to the ACMG 2015 guidelines. The results clearly indicate that the G724S mutation is pathogenic (Table 2).

### Protein structure modeling and analysis

Amino acid substitutions may affect protein function by interfering with protein folding, functional flexibility, and interfaces required for functional multimerization and protein-protein interactions. To help understand the underlying basis of the G724S substitution, we analyzed protein structures of the wild type as well as models of the G724S mutant.

**Fig. 3:**
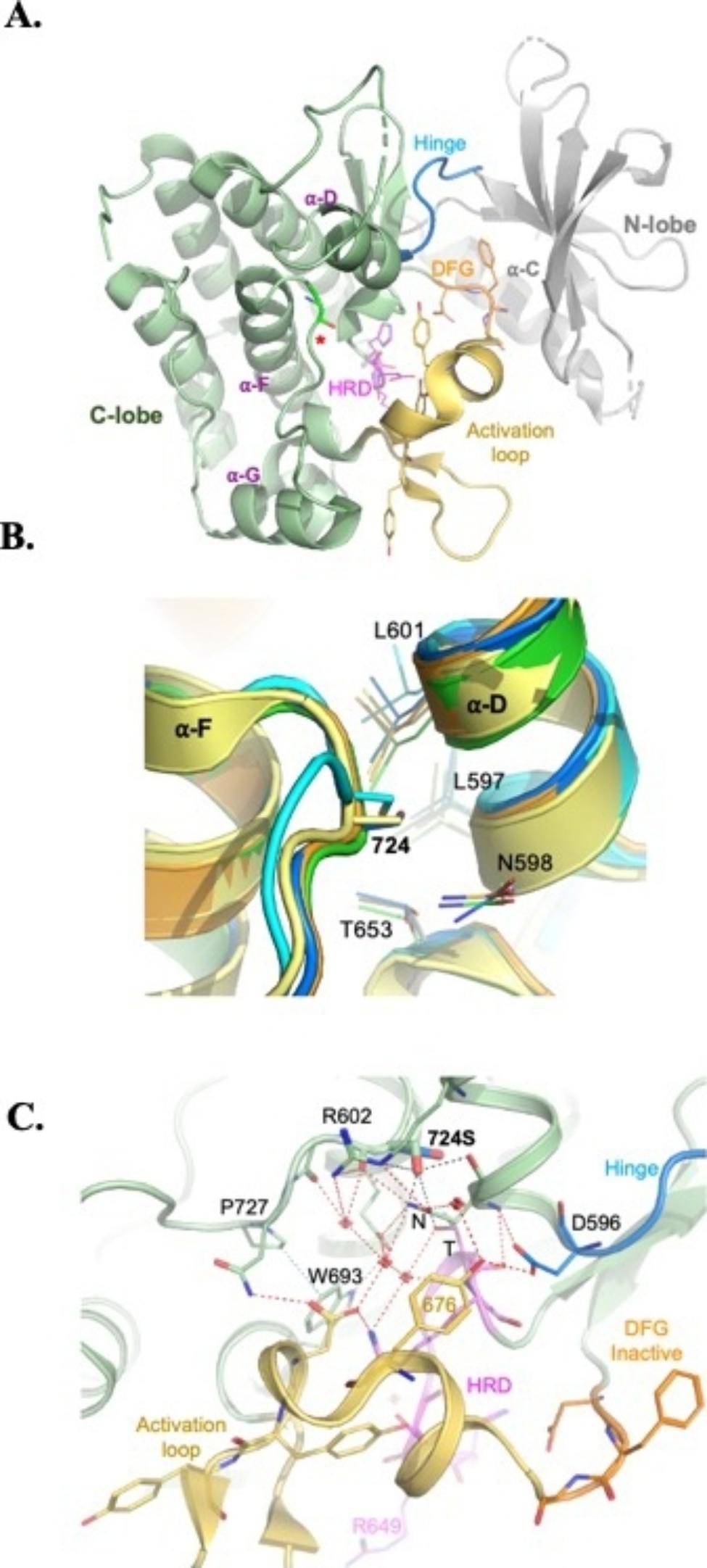
**Structural analysis of the G724S variant. (A)** An overall view of the NTRK1 protein in its inactive conformation (PDB: 4GT5). The two lobes, N- and C-terminal are labeled and colored with green (C-lobe) and grey (N-lobe). Hinge connecting both lobes (blue), the activation loop (yellow), the DGF motif (orange), and the HDR catalytic residues (magenta) are shown. G724 (red asterisk) is shown in stick representation. **(B)** Closeup view of 724 position and alignment of structures (< 1Å RMSD) containing various substitutions at this position: Ala (yellow, PDB: 5E1S), Cys (cyan, PDB: 7FEH), active form of NTRK1 (orange, PDB: 5JFX), inactive NTRK1 (green, PDB: 4GT5), and Alphafold predicted model of Ser at 724 (blue). Shown in stick representation are L601, L597, and T653, which form a hydrophobic core. **(C)** Closeup view of the kinase core of G724S mutant showing the polar network (dashed lines) that regulates conformational switches. W693 and P727 π-packing is shown (blue dashed line). 724 S is modeled in two conformations (blue and green). Red crosses represent water molecules found in the crystal structure (PDB: 4GT5). 724 S may interfere with the closest residues R602, N598 (N), and T653 (T) of the catalytic loop

NTRK1 comprises N-terminal and C-terminal lobes (N/C-lobes) connected by a flexible regulatory hinge. An additional and critical regulatory loop is the activation loop comprising tyrosine residues that are autophosphorylated during kinase activation. Upon activation, an Asp-Phe-Gly (DFG) motif, at the base of the activation loop, flips to allow ATP binding. The inactive conformation of DFG motif is in a more favorable state, and flipping into the active conformation requires large-scale movements around the active site (comprising His-Arg-Asp (HRD) motif) (reviewed in [15]) (Fig. 3A).

**Table 2:** In silico analysis and ACMG criteria of c.2170 G>A mutat

Software	Prediction result
COBALT	Conserved
Gremlin	Conserved
Polyphen	Probably damaging
Mutation Taster	Disease causing
PhD-SNP	Disease-related polymorphism
SIFT	Predicted to be damaging
Clinvar	Pathogenic
**ACMG 2015 Criteria**	**Reference (evidence)**
Variant located in critical domain	In the kinase domain, [16, 17].
Extremely low frequency	ExAC: 0.00002, gnomAD: 0.00001
Detected in trans with pathogenic variant	Reported before in [18]
Cosegregation with disease in multiple affected family members in a gene known to cause the disease.	Two family members with homozygous c.2170G > A (p. G724S) mutation are affected (Figs. 1 and 2).
Missense variants are a common mechanism of disease	Reported before in [4, 11]
Multiple lines of computational evidence support a deleterious effect on the gene or gene product.	Yes, as shown above in this table
Patients phenotype or family history is highly specific for a disease with a single genetic etiology	Yes, as described throughout the paper
Reputable source recently reports variant as pathogenic	Yes, https://clinvarminer.genetics.utah.edu/submissions-by-variant/NM_002529.4%28NTRK1%29%3Ac.2170G%3EA%20%28p.Gly724Ser%29

G724 resides within a loop connecting α-F and α-G of the regulatory C-lobe, and is at the interface between two helices α-F and α-D (Fig. 3A). A substitution of glycine to a larger amino acid at this position could result in a steric clash and potentially lead to protein misfolding. However, Alphafold structure modeling [13] of the G724S variant shows that the larger side chain of serine can still be accommodated without interfering with protein folding (Fig. 3B). Likewise, related tyrosine kinases that preserve the NTRK1 fold, such as Insulin Receptors (INSR) and Epithelial discoidin domain-containing Receptors (DDR1/2), contain comparable size amino acids, alanine or cysteine respectively, at this position (Fig. 3B). Accommodating the larger side chains of cysteine or serine appears to induce a slight movement in α-D by an average of ~ 0.9Å as measured among the various structures (at Cα of L601 or L597) (Fig. 3B). Therefore, it is unlikely that the larger size of serine side chain causes protein misfolding. However, and unlike the minor effect of its larger volume, the negative charge of the hydroxyl group of serine could impose more impact on core hydrophobic-packing and on polar-interactions. In one conformation similar to that of DDR1 Cys (Fig. 3B), the hydroxyl of serine could disrupt a critical hydrophobic core (L601, L597 and T653-methyl group) between α-D and α-F, which could cause protein misfolding. In an unrelated previous study, we noticed such and effect when substituting Cys with Ser within a similar hydrophobic vicinity (leu, Ile, and Val), which resulted in an insoluble enzyme. However, when substituting Cys with Ala or Val in this case, the enzyme was soluble and fully functional (not shown).

Alternatively, a second conformation for the hydroxyl of serine (Fig. 3C) may interfere with the polar-interaction-network at the core of C-lobe. This network critically regulates kinase activity by regulating the flexibility of α-D helix, conformational switch of DFG-motif, the activation-loop and the W693-P727 interaction at its base, as well as the flexibility and accurate positioning of the HDR catalytic loop (Fig. 3C).

Defining the underlying molecular mechanisms of the deleterious structural effects of the G724S mutation requires detailed biochemical and structural analysis, which are beyond the scope of this work.

### A novel mutation identified in SCN9A gene of CIP family

For AP05 family, clinical examination revealed that the proband had CIP without anhidrosis. In order to elucidate which mutation causes the disease in this family, we performed Whole exome sequencing (WES) for the proband. Our results confirmed the presence of a novel c.901 A > T mutation that creates a stop codon at lysine 301 (p.K301*) in *SCN9A* (Fig. 2G). To further validate the causation relationship between the identified mutation and family phenotypes, we did segregation by Sanger sequencing the gene area flanking the mutation position. Our analysis revealed that the parents and an unaffected brother were heterozygous (A/T) for the mutation (Fig. 2H), while our proband and his affected sister were homozygous (T/T) for c.901 A > T mutation (Fig. 2G). Altogether, our results demonstrate that the novel c.901 A > T mutation is the variant that causes CIP in AP05 family.

## Discussion

Individuals with CIP suffer from accumulation of injuries, fractures, infections, and bruises affecting their life expectancy [11]. Thus, early genetic diagnosis and management of pain loss patient could help in enhancing their life quality. In this research we aimed to detect the mutations that cause CIP, and CIPA in five affected unrelated Palestinian families.

In our study, we found two mutations in *NTRK1* gene in CIPA probands. The first mutation (c.1860-1861insT ) is in exon 15. This insertion generates premature termination of NTRK1 protein. The termination affects the protein function, because it occurs in the intracellular domain of NTRK1 protein which contains a Juxta membrane region, a tyrosine kinase domain, and a short carboxy terminal tail [4]. The phosphorylation of the intracellular domain in response to NGF binding is important for intracellular signaling since the phosphorylated tyrosine residue in NTRK1 cytoplasmic domain form a binding site for downstream signaling molecules [4]. Premature termination due to (c.1860-1861insT ) mutation leads to the loss of the tyrosine residues Tyr-670, Tyr-674, and Tyr-675 that form an autophosphorylation site [17]. In addition to these phosphorylation sites, other tyrosine residues such as Tyr-751 and Tyr-785 are important for NTRK1 functionality [17]. For example, Tyr-751 is part of the consensus sequence motif YXXM, which interacts with phosphatidylinositol-38 kinase, and Tyr-785 that is located at 15 amino acid from the carboxy-terminal tail is involved in the biological activities of EGF, and CSF-1 receptors [17].

In addition to our current study, in which all patients were Bedouins, the c.1860-1861insT mutation was also reported in other studies that involved Bedouins [6, 11], which confirms that this is a founder mutation that segregates in the Bedouin community.

The second mutation that we have identified in *NTRK1* gene of CIPA patient was c.2170G > A (p. G724S), that occur in exon 16. The mutation is a missense mutation that replaces glycine number 724 with serine. The mutation occurs in the conserved region in the NTRK1 C-lobe that has both regulatory and catalytic roles, and is particularly close to the catalytic core of the kinase domain near the activation loop, the DFG motif and the HRD catalytic loop (reviewed in [15]). Most probably, the hydroxyl of serine may have interfered with one or both essential core structural features: hydrophobic packing and functional polar-network at the kinase-core, which affects critical conformational switches. Our in silico analysis support a deleterious and pathogenic effect for this mutation. Moreover, the c.2170G > A mutation was previously reported in two studies [9, 12]. In one study, the mutation was revealed in a whole exome sequencing study that involved consanguineous Palestinian and Israeli Arab family with suspected neurogenetic disorder [12]. In the other study, the mutation was identified in a Jordanian study that included 7 CIPA patients [9]. The identification of the same founder mutations in Palestinian and Jordanian families is expected, since more than 50% of the Jordanian community has Palestinian roots.

Most of the mutations that cause CIP without anhidrosis fall in *SCN9A* gene [1]. But because mutations in SCN9A were not reported in our region, we did WES for the patient with CIP and without anhidrosis. Our analysis revealed a novel mutation in exon 7 in *SCN9A* gene (c.901 A > T). This change creates a stop codon at position 301. This mutation is close to a previously reported mutation (c.1124delG) which leads to protein synthesis termination in the sixth transmembrane domain in the Nav1.7 channel [19] that regulates sodium ions flow from the exterior of the cell to its interior, through central pores and generating action potential in nociceptor neurons [20]. Consequently, bi-allelic mutations in *SCN9A* gene are known to form non-functional Nav1.7 channels and failure to generate pain signals.

Although CIP and CIPA, are considered to be rare disorders, the frequency of the disease seems to be much higher in the Palestinian population than in other populations. As noticed from all family pedigrees included in this study, all families are consanguineous families. This was the case too in CIPA Jordanian and Saudi families studied before [9, 21]. In Palestine, consanguinity seems to represent one of the highest in the world [22]. Several studies showed that high consanguinity is related to higher frequency of rare diseases in rural communities like the Bedouin communities [23]. This was very obvious in our study in which three out of the five participating families were Bedouin families. This was also the case in other studies were CIP cases were also in Bedouin families [6, 11]. Moreover, our study as well as other studies confirmed the presence of the same founder mutation in different Bedouin communities residing in different geographical area which confirms that Bedouins in the Palestinian society belong to the same ancestors. These findings support the need of genetic counseling about CIP and other rare diseases that circulate in such communities in order to prevent the spread of fatal and difficult diseases. This claim is supported by the fact that during the course of our study three couples from the same tribe were referred to us to test for the c.1860-1861insT mutation before getting married. Two out of the three couples were heterozygous for this mutation.

In addition to genetic counseling, early genetic diagnosis is very important because it can prevent a lot of unpleasant complications [19]. This can be achieved by providing the affected family with the knowledge and preventive care instructions needed to deal with the affected siblings [24]. Also, orthopedic doctors need to deal with these cases with more care and regular follow up in order to prevent any complications at the level of bone injuries, which are common between CIP patients [6]. The early and regular medical interference, such as radiological imaging after injury, revealed to be the best choice in dealing with CIP cases, and improve the health condition for the affected individuals [6].

## Conclusion

This study aimed to investigate the mutations that cause CIP, and CIPA in the Palestinian community. Our study identified two mutations in *NTRK1* gene in Palestinian CIPA unrelated families, and a novel mutation in SCN9A gene that causes CIP without Anhidrosis. In conclusion, more studies are needed to detect other possible mutations that cause CIPA and CIP in our community. This will help in building a protocol for genetic testing of affected individuals. Such a protocol will make the diagnosis process and genetic counselling more efficient, faster and cheaper for affected families. More public awareness about CIP and CIPA in our community and between medical care providers is needed. In addition, the importance of genetic testing and genetic counselling is very important to increase the life quality and expectancy of the affected individuals and to prevent the spread of such a deadly disease.

## Electronic supplementary material

Below is the link to the electronic supplementary material.

Supplementary Material 1

Supplementary Material 2

## Data Availability

All data generated or analyzed during this study ar included in this published article and its supplementary information files.
