# Supplementary material for: Identification of founder and novel mutations that cause congenital insensitivity to pain (CIP) in palestinian patients
Source: BMC Med Genomics. 2023 May 30;16:120. doi: 10.1186/s12920-023-01544-5 (PMC10228059; doi:10.1186/s12920-023-01544-5)
Supplement: Supplementary file 1 — Supplementary Material 1 [file 12920_2023_1544_MOESM1_ESM.pptx]

## Slide 1
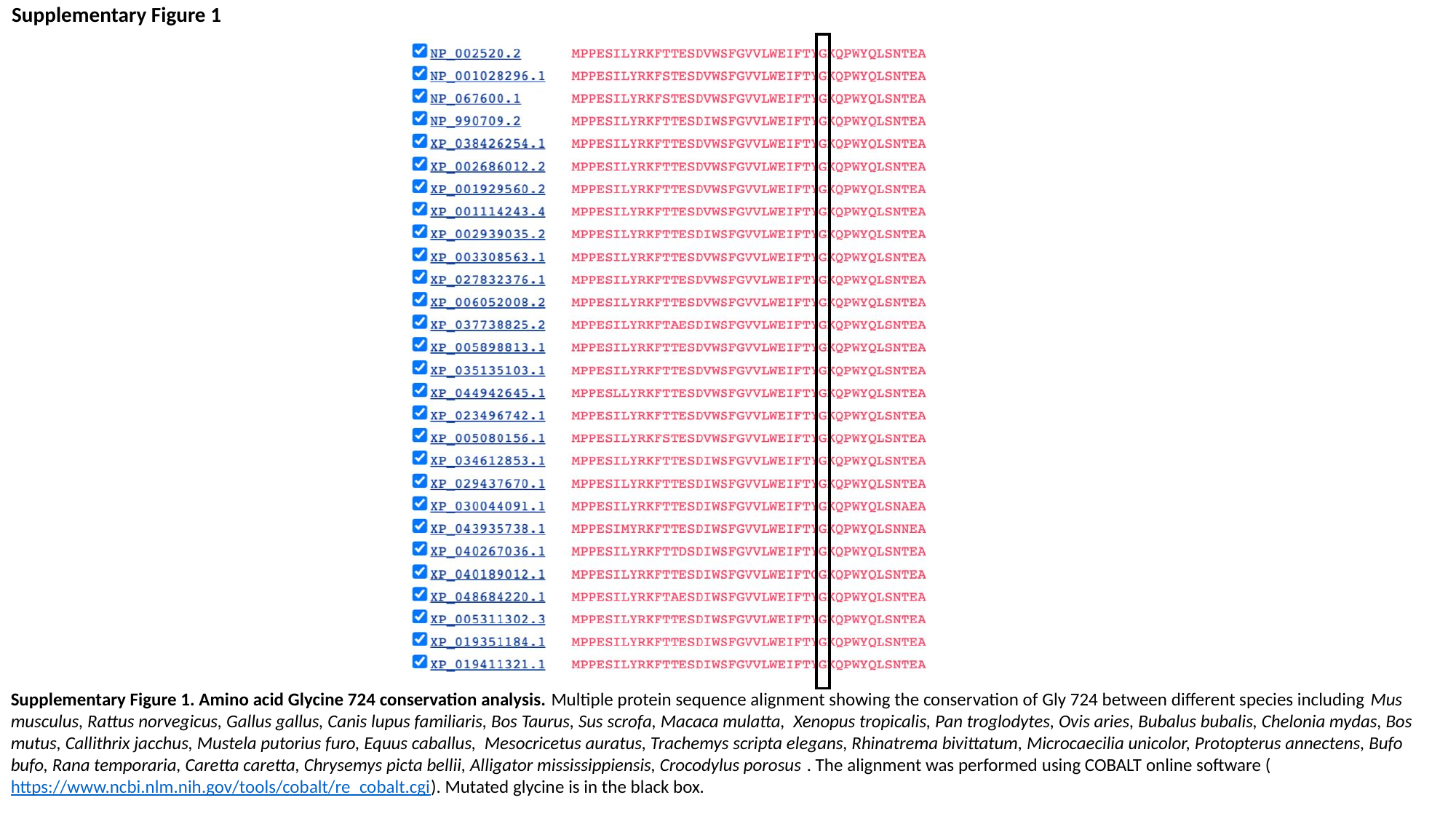

Supplementary Figure 1
Supplementary Figure 1. Amino acid Glycine 724 conservation analysis. Multiple protein sequence alignment showing the conservation of Gly 724 between different species including Mus musculus, Rattus norvegicus, Gallus gallus, Canis lupus familiaris, Bos Taurus, Sus scrofa, Macaca mulatta, Xenopus tropicalis, Pan troglodytes, Ovis aries, Bubalus bubalis, Chelonia mydas, Bos mutus, Callithrix jacchus, Mustela putorius furo, Equus caballus, Mesocricetus auratus, Trachemys scripta elegans, Rhinatrema bivittatum, Microcaecilia unicolor, Protopterus annectens, Bufo bufo, Rana temporaria, Caretta caretta, Chrysemys picta bellii, Alligator mississippiensis, Crocodylus porosus . The alignment was performed using COBALT online software (https://www.ncbi.nlm.nih.gov/tools/cobalt/re_cobalt.cgi). Mutated glycine is in the black box.
