# Supplementary material for: Identification of founder and novel mutations that cause congenital insensitivity to pain (CIP) in palestinian patients
Source: BMC Med Genomics. 2023 May 30;16:120. doi: 10.1186/s12920-023-01544-5 (PMC10228059; doi:10.1186/s12920-023-01544-5)
Supplement: Supplementary file 2 — Supplementary Material 2 [file 12920_2023_1544_MOESM2_ESM.docx]

| Exon(s) | Forward Primer | Reverse Primer | Product size |
| --- | --- | --- | --- |
| 1 | 5’-GCGGCTGGGTCTTTAACA-3’ | 5’-CATCCAGGCACTGACCATAA-3’ | 520 bp |
| 2+3 | 5’-TGGCATGTGCATGTGTATTG-3’ | 5’-CATTAGCAGCCCAAGTCTGG-3’ | 605 bp |
| 4 | 5’-CACCCCACCATCTACACACA-3’ | 5’-CACTGGCATGCACATAGTCC-3’ | 377 bp |
| 5+6 | 5’-TCTGTGTCCTCCCTTTCACC-3’ | 5’-CTGAGCCCTGTGGGTCTAAA-3’ | 820 bp |
| 7 | 5’-ACATTCTCTCCCACCCCTCT-3’ | 5’-GCAGCTGTGGAGACACACAT-3’ | 396 bp |
| 8 | 5’-CCTGTGGGGCTGTGACTTAT-3’ | 5’-ACCAGGCACTCCAATTTCTG-3’ | 701 bp |
| 9+10+11 | 5’-CGTCCCATGAAGGAATGAGT-3’ | 5’-ACACTCACAAGCCTCACAGC-3’ | 904 bp |
| 12 | 5’-TCAGTCTCTCCCCTGCAAGT-3’ | 5’-ACATGGTTTGGGCTAGCTGT-3’ | 482 bp |
| 13+14 | 5’-GGGGTGCAGGTTGAATTTTA-3’ | 5’-GTGTCTCCCCTTGGTTTGAA-3’ | 740 bp |
| 15 | 5’-CCAGTCTCCTCTCCCATCAC-3’ | 5’-TGAATCTCAGACCCATGCAG-3’ | 458 bp |
| 16 | 5’-TGAACCACCGAGCTTGTGTA-3’ | 5’-CACAGTGGAGGGGACACAGT-3’ | 426 bp |
| 7 | 5’-ACAGCTGTTCATGGGAAACC-3’ | 5’-TGACCCAAATTCACACTGTAGC-3’ | 253 bp |

**Supplementary table 1**

Supplementary Table 1: The table shows the sequence of forward and reverse primers used to sequence all *NRTK1* exons and exon 7 of *SCN9A* gene.
